# An objective measurement approach to quantify the perceived distortions of spectacle lenses

**DOI:** 10.1038/s41598-024-54368-3

**Published:** 2024-02-17

**Authors:** Yannick Sauer, David-Elias Künstle, Felix A. Wichmann, Siegfried Wahl

**Affiliations:** 1 University of Tübingen, Tübingen, Germany; 2 Carl Zeiss Vision International GmbH, Aalen, Germany; 3 Tübingen AI Center, Tübingen, Germany

**Keywords:** Human behaviour, Visual system, Optics and photonics

## Abstract

The eye’s natural aging influences our ability to focus on close objects. Without optical correction, all adults will suffer from blurry close vision starting in their 40s. In effect, different optical corrections are necessary for near and far vision. Current state-of-the-art glasses offer a gradual change of correction across the field of view for any distance—using Progressive Addition Lenses (PALs). However, an inevitable side effect of PALs is geometric distortion, which causes the *swim effect*, a phenomenon of unstable perception of the environment leading to discomfort for many wearers. Unfortunately, little is known about the relationship between lens distortions and their perceptual effects, that is, between the complex physical distortions on the one hand and their subjective severity on the other. We show that perceived distortion can be measured as a psychophysical scaling function using a VR experiment with accurately simulated PAL distortions. Despite the multi-dimensional space of physical distortions, the measured perception is well represented as a 1D scaling function; distortions are perceived less with negative far correction, suggesting an advantage for short-sighted people. Beyond that, our results successfully demonstrate that psychophysical scaling with ordinal embedding methods can investigate complex perceptual phenomena like lens distortions that affect geometry, stereo, and motion perception. Our approach provides a new perspective on lens design based on modeling visual processing that could be applied beyond distortions. We anticipate that future PAL designs could be improved using our method to minimize subjectively discomforting distortions rather than merely optimizing physical parameters.

## Introduction

The natural decline in the eye’s accommodative capabilities makes it progressively harder to focus on close objects. This natural aging process results in the eye’s lens becoming less flexible, losing its ability to change shape. Approximately 2 billion people worldwide suffer from this condition, termed *presbyopia*^1,2^; without optical correction, all adults would experience blurry close vision starting in their 40s.

Presbyopes can use reading glasses for sharp near vision, which have to be taken off for looking at distant objects—in the case of preexisting corrections, this requires constant switching between two pairs of glasses. The convenient solution combines both lenses: the near correction at the bottom and the far correction at the top of each lens^3^. Those so-called bifocal lenses, however, cannot offer correction for intermediate distances and show a visible, distracting border at the transition between both lens areas. This edge in the lens is also considered to create a stigma of glasses for “old people”. An obvious refinement is a smooth transition between the near and far areas by gradually changing the curvature of the lens surface. These Progressive Addition Lenses (PALs) became available in the second half of the 20th century through technical advances in manufacturing technologies^4,5^. Today, PALs are the state-of-the-art lens in presbyopia correction.

**Figure 1:**
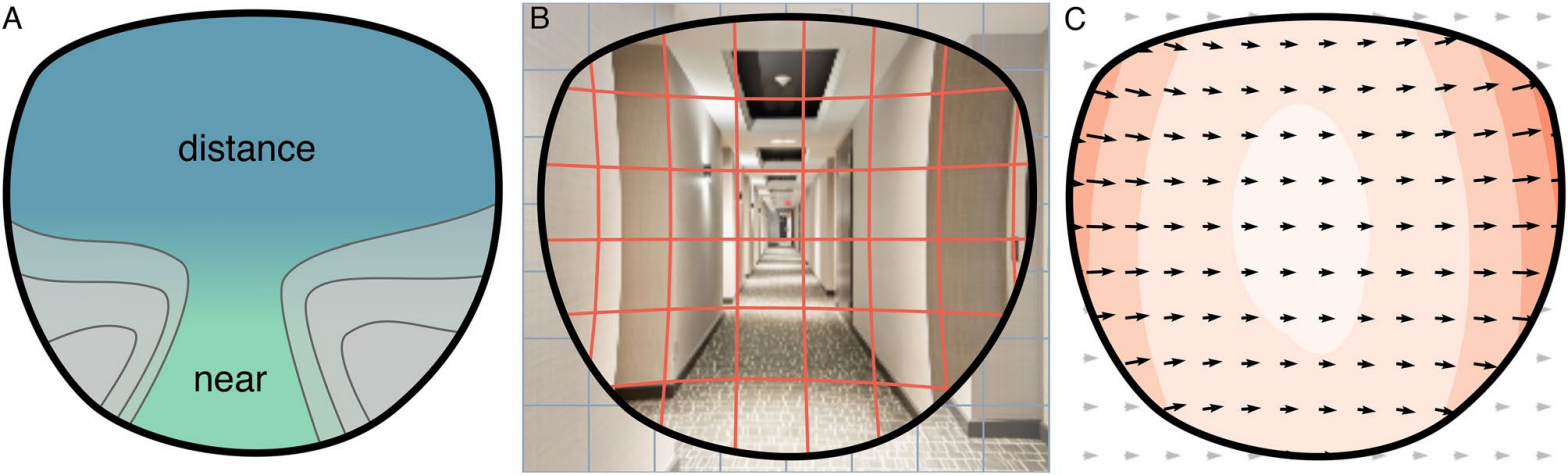
Progressive Addition Lenses (PALs). (**A**) The upper lens area is designed for far vision, with an optical power fitting the far refraction of the wearer. The optical power increases vertically towards the near area, which offers additional power for focusing close objects. The gradient in power will always lead to lateral astigmatism. (**B**) Optical distortions of PALs change the size and orientation of objects. Vertical and horizontal edges in a typical indoor environment appear curved. (**C**) Perceived motion during a horizontal head movement. The retinal motion pattern—optic flow—is altered by optical distortions. Points in the visual field move along curved trajectories instead of straight lines. Lens distortions increase towards the periphery leading to an increase in optic flow speed (illustrated by the heat map in the background). The unnatural distorted optic flow pattern can be perceived as an unstable movement of the environment (swim effect).

Even though there has been great progress in improving PALs, the gradual increase in optical correction between far and near areas will always lead to unwanted optical errors, so-called aberrations. This causes blur or degradation of sharpness in some areas of the lens. Another aberration is geometric distortion, a variation in the magnification across the visual field, leading straight lines to appear curved when looking through the lens (Fig. 1). Sadly, for PALs, it is physically impossible to reduce aberrations to zero, as stated by the Minkwitz theorem^6–8^. What lens designers and manufacturers can do, however, is to *change the distribution of aberrations* in the field of view—attempting to find subjectively more benign patterns of aberrations across the visual field.

This flexibility in shifting the aberrations to different areas in PAL design is used to develop specific PALs for tasks like driving or office work. Such PALs show reduced blur in task-relevant areas, inevitably accompanied by increased blur in other areas, however.

How to optimize the design for distortions *in general* is an open question, however, because their influence on visual perception is poorly understood. The swim effect, a phenomenon of unnatural or unstable perception of the environment during head or eye movements, causing instability, dizziness, tripping, and nausea^8–11^, is at least partly caused by geometric distortions. It is unclear how those effects scale with the physical distortion of the lens and its distribution across the visual field.

Understanding and quantifying the influence of distortions on human perception can—in future applications—enable PALs with reduced distortion-induced discomfort. To our knowledge, this study presents the first rigorous measurements of perceived distortions of PALs.

The main influence on PAL distortions is the optical correction in the far and near area, the optical power measured in diopters. The correction in the far area, the spherical power *Sph*, can be positive or negative (correction for hyperopia or myopia, respectively); in the near area, the additional power *Add* usually increases with the age of the wearer, since the eye can accommodate less and less by itself. The optical power changes progressively from the upper far area to the near area below, allowing vision at intermediate distances. Depending on the sign of *Sph*, the general shape follows a pincushion or barrel distortion^12^, i.e., curving straight lines more inwards or outwards. Additionally, distortions show asymmetry between near and far areas, influenced by *Sph* and *Add*. Relating *Sph* and *Add* to perceived distortion builds a foundation for understanding PAL-induced discomfort.

Our suggested measurement is a psychophysical scale, quantifying the relative change of perceived distortion for different combinations of *Sph* and *Add*. This scale indicates how much distorted a lens feels if *Sph* or *Add* changes by a certain amount of diopters. We present distortions of PALs of different near and far correction in a virtual reality (VR) simulation. Aberrations of ophthalmic lenses have been studied previously using simulations in screen-based or VR set-ups^13–16^, which allow greater control over the stimulus while, in the case of VR, still allowing natural behavior. In our experiment, subjects can move freely in a virtual indoor environment, inducing distorted motion perception under natural self-motion. To study the influence of geometric distortions independent from other typical aberrations of PALs, we simulate only geometric distortions, not the blur caused by other lens aberrations.

Each trial consecutively presents three out of eleven simulated PAL distortions of various *Sph* and *Add*. Subjects responded which distortions appeared more similar (1 & 2 or 2 & 3). This ordinal data is used to fit the subject’s perceptual distortion scale with ordinal embedding methods^17^. Unlike other studies about PAL distortions, our embedding method results in an objective scaling function, which can quantify the relative influence of different lens parameters.

In contrast to screen-based distortion studies (^18–23^), VR technology allows considering stereo and motion perception like the swim effect. It minimizes the lab-to-reality gap—increases ecological validity^24^—by recreating actual lens distortions in realistic environments in which observers can move and experience visual consequences of their own actions.

In summary, we measure the perception of geometric distortions of PALs in a realistic VR environment with natural head movements—decoupled from other typical optical aberrations. Using an ordinal comparison-based experimental paradigm in combination with an analysis by an embedding algorithm, we derive their perceptual scales individually for every observer. Our statistical modeling predicts perception across subjects well, allowing a potential application of our method for improving spectacle lenses by reducing perceived lens distortions for a generic observer.

## Methods

### Subjects

Subjects wearing spectacle lenses might already be habituated to certain distortions over the often long time having worn them. Thus only emmetropic subjects were included in the experiment to exclude this as a possible confounding factor; we assessed acuity of all subjects with the 6/6 Snellen chart. Seven male and seven female participants (mean age 24.6 years; SD 4.0 years) were confirmed not to have any known ocular diseases. One of the male subjects decided to discontinue the experiment because of VR sickness; therefore, the results of 13 subjects were analyzed. The study followed the principles of the Declaration of Helsinki and was approved by the ethical board committee of the University of Tübingen (439/2020BO). Informed consent was obtained from all participants before the measurements.

### Stimuli

**Figure 2:**
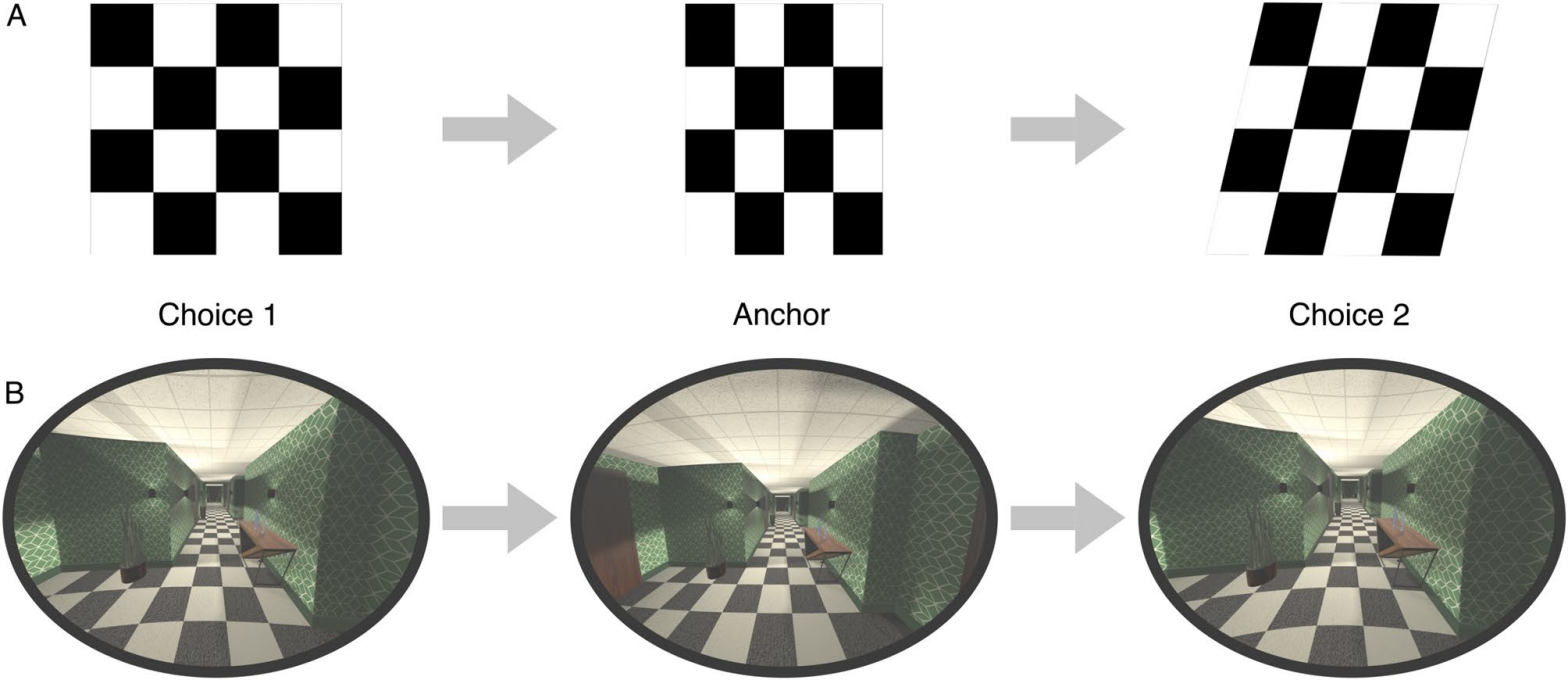
The triplet paradigm of the experiment presents three distortion stimuli consecutively in each trial: choice 1, anchor, and choice 2. The task for subjects was to answer if the first distortion (choice 1) or the last distortion (choice 2) is more similar to the second distortion (anchor). This task is equivalent to asking which of the two transitions between distortions seems smaller. (A) The checkerboard pattern with simple distortion transformations demonstrates the task during the initial training phase. (B) The experiment simulated PAL distortions in a virtual indoor environment. While the different distortions were presented, subjects could look around freely. Distorted motion perceived during dynamic behavior is expected to cause an unnatural or unstable perception of the environment.

Subjects looked through simulated spectacles in a 3D-modelled hallway using an XTAL VR headset (VRgineers Inc, Prague, Czech Republic). The headset’s horizontal FoV of 140 degree allows realistic simulation of spectacle lens distortions, affecting the part of the FoV covered by real spectacle lenses. The virtual environment was designed to replicate a scenario where distortions are visible clearly for PAL wearers: an indoor environment with many horizontal and vertical edges, shown in Fig. 2.

Distortions of ten different PALs were included in the experiment. The far refraction ranged from $-5\,\hbox {dpt}$ to $5\,\hbox {dpt}$ in steps of ${2.5}\,\hbox {dpt}$. For each of those five *Sph* values, two lenses with *Add* power 1 dpt and 3 dpt were used. All 10 lenses had the same PAL design (ZEISS Smart Life). An additional undistorted condition was included for reference. The distortions were precalculated using ray tracing based on the lens surface data provided by the manufacturer^25^. The precalculated distortions are represented as horizontal and vertical displacement of image plane coordinates; the displacement vectors are stored pixel-wise as two color channels of a texture. In the Unity game engine, the texture is used as an input to transform the rendered image by performing a coordinate transformation in a fragment shader. The procedure is performed independently for left and right eye cameras. In our experiment, the same *Sph* and *Add* corrections were used for both eyes, resulting in horizontally mirrored distortions.

Similar to the edge of a spectacle frame, we show an ellipse-shaped mask in the FoV that separates the distorted “lens area” from the undistorted periphery. The XTAL VR headset has an FoV larger than typical spectacle lenses, which makes it possible to simulate a typical inner frame size of ${54}\,\hbox {mm}$ by ${28}\,\hbox {mm}$ with an ellipse of ${116}^{\circ }$ inner width and a height of ${80}^{\circ }$. This frame ellipse was rendered on the image for each eye. The monocular FoV is limited in the nasal direction to ${45}^{\circ }$. Therefore, the ellipse is cut off in the nasal direction. The combined binocular perception shows a full ellipse. The ellipse thickness was ${2}^{\circ }$ in visual angle.

### Experiment procedure

The experiment used a triplet paradigm, presenting three distortion stimuli in each trial. The three distortions—choice 1, anchor, and choice 2—were all sampled from the predefined set of 11 stimuli (10 distortions and undistorted). Presentation time for each distortion stimulus was ${2}\,\hbox {s}$. Between stimuli, a ${0.2}\,\hbox {s}$ transition was fading the image first to black and then to the next stimulus. Subjects had to judge the similarity of perceived distortions. They answered with a button press on a controller, which distortion—choice 1 or choice 2—appeared more similar to anchor. An alternative but equivalent instruction asks for the smaller transition: from choice 1 to the anchor or from the anchor to choice 2. In the experiment, both variants were used with the subjects.

Subjects were familiarised with this experiment procedure in multiple training phases. To clarify the experimental paradigm, in the first phase, stimuli were only checkerboard patterns distorted with clearly distinct transformations to make it easy for subjects to distinguish the distortions. One example is shown in Fig. 2. In phase two, the stimuli showed the 3D environment of the main experiment but with trivial distortion combinations (including easy-to-perceive distortions of lenses with *Sph* ${8}\,\hbox {dpt}$ or ${5}\,\hbox {dpt}$ together with clearly less distorted lenses). During those two training phases, a green or red colored background at the end of each trial gave feedback to the subjects if their answer was as expected (choosing the two strongly distorted or the two clearly less distorted lenses as more similar).

In the last training phase, the distortion combinations were similar in difficulty to the main experiment. No feedback was given after the subject’s answer. This training phase ensured that all subjects had learned the triplet task and were familiar with the magnitude of (real-world) lens distortions used in the subsequent experiment.

The experiment was performed in a seated position while wearing the VR headset. Subjects could move and look around freely. To induce the swim effect, subjects were encouraged to move their head. This was done by tracking head movements during training phases 2 and 3 and only continuing the trials when subjects moved their head.

The perceived differences between some distortion stimuli can be small, possibly causing frustration. To increase motivation, the experiment included $20\%$ of trivial trials, where one or two of the stimuli were exaggeratedly distorted (*Sph* ${8}\,\hbox {dpt}$) and the other was slightly or not distorted. Additionally, these trivial trials provide some baseline validation of subject performance because the misfitting stimulus of the three distortions is obvious and should never be chosen when following the experiment instructions correctly. In total, every subject did 413 trials, of which 83 were trivial. Each triplet combination of the 11 stimuli was presented twice, with flipped order of choice 1 and choice 2 in the second presentation. The trial order was randomized. Subjects could repeat the presentation of a trial any number of times if they felt too uncertain to respond, which might be necessary when there is a great similarity between choice 1 and choice 2 (the number of repetitions per subject can be found in Supplementary Fig. S1 online).

During the experiment, headset tracking data was recorded for subsequent analysis of head movement behavior. The SteamVR 2.0 tracking system (Valve Corporation, HTC)^26^ was used with four base stations located around the participants’ seating positions. Gaze data were captured with 120 Hz sampling frequency using the VR headset’s included video-based eye tracker, accessed via the VRGineers XTAL Unity Plugin (version 2.08) and VRGineers XTAL runtime 3.0.0.77. For calibration, the manufacturer’s 5-point calibration was used.

### Data analysis

#### Psychophysical scale

The subject’s perceived distortion was estimated from their trial responses (choice 1 or 2 is more similar to anchor) using so-called ordinal embedding methods^17^. These methods estimate a psychophysical scale that assigns coordinates to each stimulus so their distances agree with the subject’s similarity judgments. Specifically, we used the *Soft Ordinal Embedding* (SOE)^27^ algorithm, implemented in the *cblearn* Python package. The dimensionality of the scale has a great influence on how well the subjects’ responses are represented in general. We chose the lowest dimensional scale that still is a good predictor of unseen triplet responses. This predictive accuracy is approximated with a cross-validation procedure^28^. The robustness of our scale can be approximated by repeated estimates on resampled sets of responses (bootstrapping); low spread across scale samples indicates that the scale is determined well by the responses.

The perceived distortions in a scale are only a relative measure and not an absolute value—to compare scales between subjects or to create an “average observer” scale; we aligned all scales using generalized Procrustes analysis^29^. This method minimizes the Euclidean distance between scales by iterative similarity transformations (translation, scaling, and flipping in our case) towards the mean scale. After alignment, we shifted all scales such that the origin—on average—corresponds to the undistorted lens (*Sph* ${0}\,\hbox {dpt}$ and *Add* ${0}\,\hbox {dpt}$). Accordingly, the average scaling value of the distorted lens *Sph* ${5}\,\hbox {dpt}$ *Add* ${3}\,\hbox {dpt}$ has a distance 1 to the origin.

#### Head movement and gaze behavior

From tracking data of headset position and gaze direction, the individual behavior during the experiment was analyzed. Since the experiment was performed in a seated position, relevant head movements are mainly rotations. We analyzed the changes in head direction by transforming the tracked headset orientation into yaw, pitch, and roll angles (Tait-Bryan angles with order y-x-z) as illustrated in Fig. 4A. The movement velocity was computed independently for each rotation component. We calculated the mean velocity for each subject over each trial to illustrate changes in motion behavior over time. Aggregated velocity can be used to compare strategies between subjects.

Eye tracking in the VR headset is implemented independently for the left and right eye. First, the combined binocular eye gaze direction was calculated as the average of both eyes’ direction vectors for each gaze sample. We compared gaze behavior between subjects by the area covered in the visual field. To do so, the binocular gaze samples were transformed to longitude and latitude coordinates in the FoV. Then, a heatmap of individual gaze distribution was calculated with a Kernel Density Estimator of bandwidth 2 degrees as a verified upper bound of eye-tracker precision. We then calculated the solid angle of the 5% percentile, meaning the area in the heatmap, which includes 95% of the distribution’s mass.

## Results

### A one-dimensional scaling function models perception of PAL distortions

**Figure 3:**
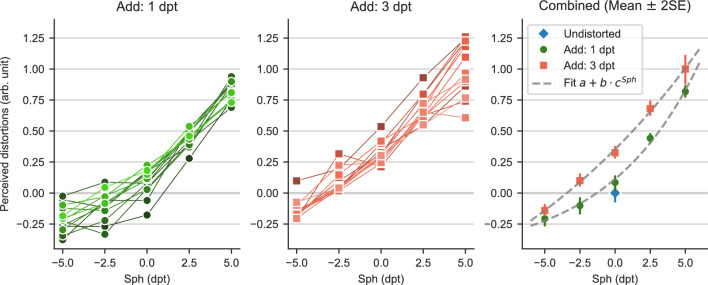
Psychophysical scaling functions of “perceived distortion” depending on the *Sph* and *Add* power of the simulated PALs. The lines in the left and middle plots show individual subjects, while the right plot shows their mean and standard error along an exponential function fit.

Although physical descriptions of PAL distortions require many parameters, the perception of lenses can deviate from this parametrization. In our experiment, we use PALs of different *Sph* and *Add*, which influence the physical distortions differently: *Sph* influences more the overall shape and strength of distortions, while *Add* introduces more asymmetry in the distortion pattern. Multidimensional perception of these multidimensional distortions seems plausible. Any low-dimensional scale provides insights into which lens parameters dominate our perceived similarity of lens distortions and whether these parameters are the same in all persons. We measured individual scales of 13 subjects from triplet responses of 11 lenses of varied *Sph* and *Add*; lenses that are judged as more similar in the responses appear closer in the scale. Indeed, all subject scales are one-dimensional (Fig. 3) and show a comparable influence of *Sph* interacting with *Add*. Additional dimensions do not increase the predictive accuracy for all subjects (see Supplementary Fig. S5). This may be regarded as surprising, given the two varied lens parameters *Sph* and *Add* and their non-linear spatial effect on distortions. Apparently, the human visual system perceives the complex PAL distortions along a single dimension only.

The perceived distortion monotonically increases with both *Sph* and *Add*. For negative *Sph* values, an increase in *Add* leads to less perceived distortions (closer to undistorted), implying a compensation of perceived distortions; for positive *Sph*, an increase in *Add* only increases perceived distortions further. All in all, if *Sph* is strong compared to *Add*, the shape of distortions is mainly influenced by the sign of *Sph*, leading to either pincushion or barrel distortions, which cause different perception as proven by the change of sign of perceived distortions (with undistorted at 0). The relation between perceived distortions and *Sph* can be modeled with an exponential fit of $a+b\cdot c^{{\text {Sph}}}$ for each value of *Add*, shown in Fig. 3 (right), illustrating a higher rate of increase for positive *Sph* compared to negative *Sph*. Fits between *Add* 1 and *Add* 3 mainly differ in the offset *a* and slope *b*, but barely in the base *c*. Close to *Sph* 0, the exponential fits show a similar rate of increase for *Sph* and *Add*. With higher correction values for *Sph*, the relative influence of *Add* decreases, indicating that for high-power lenses, *Sph* dominates distortions. The individual deviations from this exponential model do not have to be due to perceptual differences or measurement accuracy alone but can also be explained by behavior—if the distortion is perceived locally, it makes a difference where the subject looks through the lens and how they move.

### Head and gaze tracking reveals subjects’ different behavior

From reports about the swim effect—an unnatural and unpleasant percept of PAL distortions during motion—we expected that especially dynamic behavior might lead to a heightened perception of distortions and thus help subjects in discriminating the stimuli. In fact, we introduced subjects to this idea by explaining the possibility of distortions becoming apparent more clearly during self-motion. Furthermore, during the training phase of the experiment, head movement was actively enforced by our experimental design. To analyze the participants’ motor behavior in more detail regarding which kind of behavior they would choose to discriminate distortions, the tracking data of head and eye movements was analyzed. This allows for identifying the strategies subjects followed to distinguish distortions and test for the possible influence of behavior on the perception of distortions.

**Figure 4:**
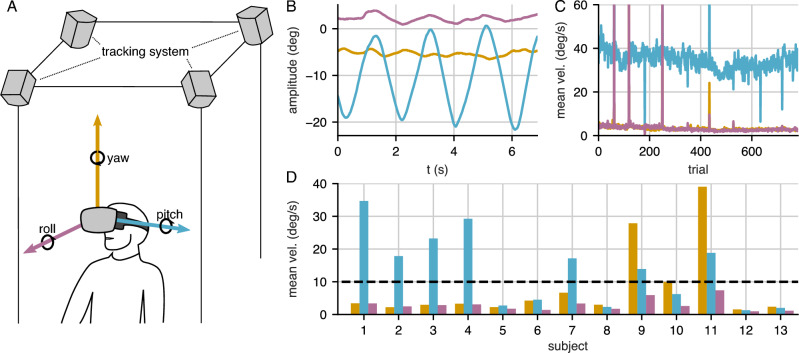
Head movements analyzed from VR headset tracking data (**A**) Tracking setup and definition of head rotation angles. Yaw, pitch, and roll were computed from the tracked orientation of the VR headset. (**B**) The three head rotation angles during one example trial. This example subject performed a continuous pitch movement (head nodding), while the roll and yaw angles stayed relatively stable. (**C**) The mean rotational velocities were calculated for each head rotation component over individual trials. Data is shown for one example subject that consistently followed the same head movement behavior. (**D**) The overview of mean angular velocities for all participants shows different head movement strategies: Some observers did not move their head, while others performed mainly a nodding (pitch) or mainly a horizontal movement (yaw).

Results of the head tracking show that subjects followed two different strategies: one group of 7 subjects performed continuous head movements, usually a nodding movement (pitch oscillation like in Fig. 4B and C, some also yaw movements, while the other group of 6 subjects did not move their head or stopped after a few trials. Since subjects usually followed the same movement type throughout the experiment (head movement over time can be found in Supplementary Fig. S2), we calculated the mean velocity over the whole experiment as shown in Fig. 4D.

**Figure 5:**
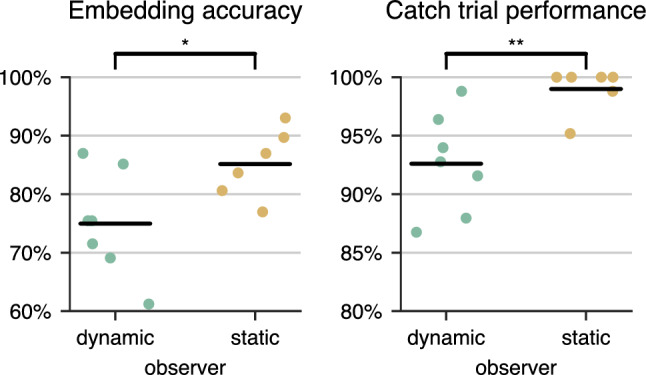
Difference in embedding accuracy and performance in the trivial catch trials between dynamic observers (head movements) and static observers (no head movements).

We grouped subjects in dynamic and static observers based on their mean head movement velocity. If the mean velocity of any of the three rotation components was higher than the defined threshold of ${10}^{\circ }\,\hbox {s}^{-1}$ the subject was classified as dynamic observer; otherwise as static observer. The mean roll rotation velocity never was higher than the threshold. Dynamic subjects primarily performed horizontal (yaw) or vertical (pitch) movements. The rotation velocity threshold was chosen in agreement with the examiners’ observation during the experiment. The static observers had to rely only on static distortion features in the scene for their comparison judgment. During the training phase, head movements were enforced; consequently, dropping this strategy during the main experiment either indicates that the movement does not convey relevant cues for the observers or that the non-moving observers were less motivated to perform well. We compared the consistency of dynamic and static observers regarding embedding accuracy and catch-trial performance. Accuracy counts the number of triplets that agree with the estimated embedding and thus measures the general coherence of responses; responses to catch-trials, however, should be unambiguous, and any error indicates a lack of concentration. There is a significantly better embedding accuracy ($p < 0.05$) as well as performance in the catch trials ($p < 0.01$) for more *static* observers using the Mann-Whitney U rank test (see Fig. 5). Consequently, it is unlikely that static observers were less motivated. Instead, for those subjects, dynamic features contributed less to the perception of distortions. This result contrasts with the expectation that especially dynamic behavior, associated with the swim effect, would give a clear cue for distinguishing distortions and more reliable results from dynamic observers.

**Figure 6:**
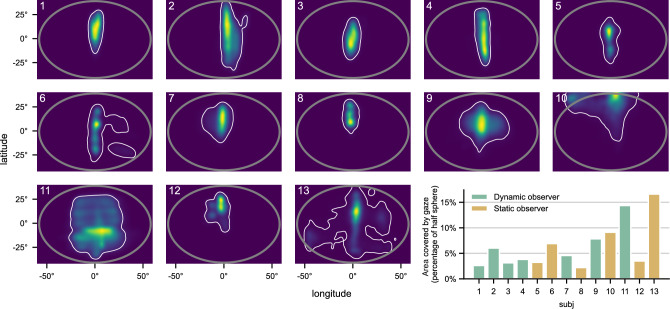
Individual gaze distribution of all observers with Gaussian kernel smoothing. Binocular gaze samples (in head-relative coordinates) of the whole experiment were combined to calculate the gaze distribution in the observers’ FoV. The grey ellipse represents the virtual frame size, which was visible as a black border during the experiment. The white contour encompasses 95% of the gaze distribution mass. The area enclosed by this contour for individual subjects is shown in the bar diagram as percentage of the half-sphere area.

The distribution of gaze in the FoV for the individual subjects is shown in Fig. 6. The gaze was mainly oriented along the center vertical axis, with variations in the latitude between individual observers. Some subjects show a high spread in gaze direction, while others stay in a more defined area of the FoV. This is reflected by the gaze area measure calculated from the individual gaze distributions. This finding suggests that some subjects, with a small gaze area, continuously fixated on the same part of PAL distortions, while others looked more at different parts of the distortion pattern. Next, we want to test if the described differences in behavior also influence the scaling of perceived distortions.

### Distortion perception may not be determined by overt behavior

PAL distortions cause a complex, spatially varying transformation of the visual space, altering static and dynamic features and therefore the perception of shape, distance, and motion. Our behavior, however, influences which visual features we perceive; for example, head movements introduce perceived motion. If the various aspects of distortion perception scale differently with the PAL distortion components, then this should be revealed by differences in the scaling function between subjects of different behavior. Especially the difference between static and dynamic observers should show how the swim effect, associated with dynamic situations, contributed to distortion perception. But also, different gaze strategies might cause differences in perceived distortion and thus in the recovered perceptual scales. Eye tracking results revealed differences in the spread of gaze. A wider area of gaze implies that subjects see a higher variability in distortions.

To statistically evaluate this possible influence of behavior on the scaling function, we used a linear model to predict perceived distortions depending on *Sph*, *Add*, and head and gaze behavior. For the two head-movement groups, we introduced a categorical variable in the model; gaze spread is modeled by the area in the field of view, covered by gaze (according to 95% KDE distribution mass, see Fig. 6). The model’s fixed effects include *Sph*, *Add*, head-movement group, gaze area, and all their interactions. The preceding Procrustes analysis aligned the subjects’ individual scales already by shifting and scaling so that random effects for slope or intercept do not have to be considered in the linear model.

To fit the linear model, we used statsmodels’s ols function in Python. *Sph* and *Add* both show significant effects on perceived distortions ($p < 0.001$). No other effects or interactions are significant. The differences in head movement and gaze behavior did not lead to significantly different perceptions of distortions, which indicates that perceptual effects of both static and dynamic features scale similarly with the amount of PAL distortions.

This result is, from a practical point of view, very good news: It implies that the general scaling of distortion perception is similar for all observers, independent of their specific and often idiosyncratic head and eye movements; different behavior does not lead to differently perceived distortions, which in turn allows a general quantification of perceived distortions for a specific PAL.

### A non-linear fit of scales predicts perception of PAL distortions

Only if we can make predictions about the distortion perception of unmeasured subjects—based on scales measured from other subjects— is there a possibility that perception models can actually be used to improve lens designs beyond individual designs. We assessed the predictive ability of three differently complex regression models in a leave-one-subject-out procedure: the models are trained on all but one subject’s scaling functions and subsequently tested on the omitted subject. We found that the perception of most subjects follows the same regularities, well captured by an exponential model.

**Figure 7:**
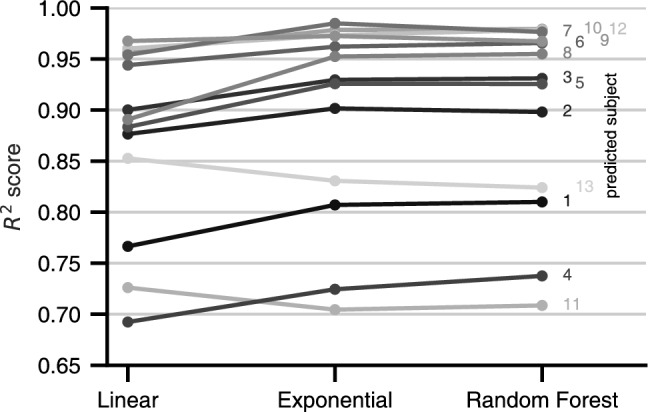
Performance in predicting a subject’s perceived distortion with models that are trained on the remaining subjects’ scales along *Sph* and *Add*. The linear and random forest models indicate baseline and ceiling performance. Exponential functions of *Sph*, grouped by same *Add*, predict similarly well as the much more general random forest model.

The assessed models include the exponential fit from Fig. 3 along with baseline and ceiling performance models to provide a reference. The baseline model is a linear regression, and the ceiling model is a random forest regressor^30^, known for excellent out-of-the-box performance in non-linear problems. $R^2$ scores in Fig. 7 show an overall high predictability in all but the baseline model, indicating that most of the subjects’ scales can be predicted accurately. Inspecting the scales with lowest $R^2$ scores (compare Supplementary Fig. S4), we see some individual differences. In the extremes, subject 1 shows a noticeably higher influence of *Add* and a more pronounced flattening in negative *Sph*, while the *Add* parameter has almost no influence in the scale of subject 13. The responses of subjects 4 and 11 during the experiment seem less consistent, resulting in higher variations of the scaling estimates. For these subjects, the collection of additional trials might improve the agreement with other subjects and, therefore, improve predictability.

For most subjects, the exponential and random forest models substantially increase prediction over the linear model, again underlining the non-linear influence of *Sph* and *Add*. The random forest, however, can use its greater flexibility with only one subject to predict the scale better—an exponential model actually seems to describe the relationship between perceived distortions and lens parameters very well.

## General discussion

We performed a VR experiment to determine a psychophysical scale of perceived optical distortions of PALs. Distortions were simulated in VR based on precalculated ray tracing of real PAL designs. A triplet paradigm was used to retrieve ordinal data of the relative perceived distance of distortions. The approach was tested with a set of PALs of varying *Sph* power (far correction) and *Add* power (near correction). In our explorative study, observers could move their heads and eyes freely to induce the perception of unnatural motion (swim effect) associated with the distortions of progressive lenses.

The scaling functions retrieved by fitting the ordinal data show a similar trend for all observers: perceived distortions increase with spherical power. For positive *Sph*, increasing the *Add* power results in stronger distortions. In contrast, the positive *Add* power in combination with negative *Sph* power reduces the perceived distortions (closer to undistorted), suggesting an advantage for short-sighted PAL wearers. For very high or very low *Sph*, the relative influence of *Add* on perceived distortions seems less relevant; close to *Sph* 0, *Add* and *Sph* seem to have a similar influence on perceived distortions. The relationship is very well modeled by a simple exponential function.

Behavior measurements of head and eye movements show that observers followed different strategies in the experiment. The most apparent strategy difference is the head movement behavior, which also leads to differences in the performance in catch trials. Some subjects moved their head continuously (nodding or shaking motion) while others kept their head stable, despite being instructed in the training of the experiment to perform head movements. This indicates that some observers might be less sensitive to perceive distortions from optic flow. This also agrees with the experience of PAL wearers: some individuals are more sensitive to the swim effect than others^11^. A speculative explanation would be that subjects who relied on head movements perceive the influence of distortions on optic flow more clearly and might suffer more from the swim effect.

However, no influence was found by head movement or gaze behavior for the psychophysical scaling function. We conclude that the global distortion pattern, not only the local distortions, influences the perception of distortions with static and dynamic features. For our virtual scene, distortions perceived from static or dynamic features scale similarly to the PAL parameters. Additionally, the scales generalize well to new observers, confirming that our psychophysical scale can be used as an objective quantification method for PAL distortions. The remaining differences between subjects’ scaling functions (for example, differences in the relative influence of *Add* power) could result from individual differences in perception or behavior. We suggest follow-up experiments focusing on increased stimulus and behavioral control. One aspect of our experiment that would be worth investigating in a more controlled setting is the significance of binocular vision for the swim effect. VR technology offers the opportunity to control binocular vision, which helps in investigating the influence of distortions on depth and surface curvature perception. It might be vital to perform binocular tests to ensure suitable participant recruitment. In any of those potential follow-up experiments, an increased number of participants would provide valuable information to identify potential patterns in the perception of observer groups, which could be included in our perceived distortion model.

Further research should investigate static versus dynamic effects in detail as well as extend our inquiry to other lens aberrations. Our study concentrates on the influence of distortions on perception. For real PALs, spherical, astigmatic, and higher-order aberrations negatively impact vision. The possible influence of those aberrations and the interaction with distortion perception could be studied in future experiments by including realistic blur in our VR simulation^31^. In more realistic viewing conditions, PAL wearers will adapt their behavior to gaze through the clear areas of the lens^32^. This development of a “head-mover” behavior^33^ should not be confused with the dynamic movement strategy of one part of our subjects. In our experiment, the subjects’ behavior reflects their strategy to distinguish differences in the distortions, while in everyday life, PAL wearers would likely follow an approach that minimizes discomfort. The gaze distribution would be determined by the current activity. For instance, during walking, the gaze would be directed downwards. In that sense, our results for behavior do not reflect the influence of PALs in everyday life but individual strategies for distinguishing between distortions.

### Conclusion

In this study, we introduced a new measurement method for determining a psychophysical scale of optical distortions. Our measurements show a high agreement between subjects, allowing predictions of PAL distortion perception in general. These results reveal the potential of using psychophysical methods for understanding the swim effect and could help to improve future optical designs of PALs. As stated by the Minkwitz theorem, a total reduction of aberrations in the lens is impossible. Design choices for a given correction power can only change the spatial distribution of aberrations. Choosing a design with a lower amount of perceived distortions can contribute to reducing distortion-related discomfort for PAL wearers and increase satisfaction. To quantify different designs for their perceived distortion, it is required to repeat our experiment to measure perception not only depending on the correction power (*Sph* and *Add*) but directly on parameters describing the possible differences in PAL designs for a given correction. With a model based on the results of this suggested experiment, an arbitrary PAL design could be quantified for perceived distortions purely based on ray-traced distortion data without testing it in an additional experiment. As a completely new approach to lens design, this perception-focused optimization might lead to a realignment of current lens design processes.

### Supplementary Information

Supplementary Figures.

## Data Availability

The dataset generated during this study as well as the analysis code is available on GitHub (https://github.com/ZeissVisionScienceLab/PAL-distortion-scaling).

## References

[1] Fricke TR (2018). Global prevalence of presbyopia and vision impairment from uncorrected presbyopia: Systematic review, meta-analysis, and modelling. Ophthalmology.

[2] Charman WN (2014). Developments in the correction of presbyopia i: Spectacle and contact lenses. Ophthal. Physiol. Opt..

[3] Letocha CE (1990). The invention and early manufacture of bifocals. Surv. Ophthalmol..

[4] Pope, D. R. Progressive addition lenses: History, design, wearer satisfaction and trends. In *Vision science and its applications*, NW9 (Optica Publishing Group, 2000).

[5] Sullivan CM, Fowler CW (1988). Progressive addition and variables focus lenses: A review. Ophthal. Physiol. Opt..

[6] Minkwitz G (1963). Über den flächenastigmatismus bei gewissen symmetrischen asphären. Opt. Acta Int. J. Opt..

[7] Sheedy JE, Campbell C, King-Smith E, Hayes JR (2005). Progressive powered lenses: The Minkwitz theorem. Optometry Vis. Sci..

[8] Meister DJ, Fisher SW (2008). Progress in the spectacle correction of presbyopia. Part 1: Design and development of progressive lenses. Clin. Exp. Optomet..

[9] Sauer Y (2022). Self-motion illusions from distorted optic flow in multifocal glasses. Iscience.

[10] Johnson L, Buckley JG, Scally AJ, Elliott DB (2007). Multifocal spectacles increase variability in toe clearance and risk of tripping in the elderly. Investig. Ophthalmol. Vis. Sci..

[11] Alvarez, T. L. *et al.* Adaptation to progressive lenses by presbyopes. in *2009 4th International IEEE/EMBS Conference on Neural Engineering*, 143–146 (IEEE, 2009).

[12] Jalie M (2020). Modern spectacle lens design. Clin. Exp. Optom..

[13] Marin, G., Terrenoire, E. & Hernandez, M. Compared distortion effects between real and virtual ophthalmic lenses with a simulator. in *Proceedings of the 2008 ACM Symposium on Virtual Reality Software and Technology*, VRST ’08, 271-272 (Association for Computing Machinery, New York, NY, USA, 2008).

[14] Rodríguez Celaya, J. A., Brunet Crosa, P., Ezquerra, N. & Palomar, J. A virtual reality approach to progressive lenses simulation. in *XV Congreso Espanol de Informatica Grafica* (2005).

[15] Nießner, M., Sturm, R. & Greiner, G. Real-time simulation and visualization of human vision through eyeglasses on the gpu. in *Proceedings of the 11th ACM SIGGRAPH International Conference on Virtual-Reality Continuum and its Applications in Industry*, 195–202 (2012).

[16] Barbero S, Portilla J (2017). Simulating real-world scenes viewed through ophthalmic lenses. J. Opt. Soc. Am. A.

[17] Haghiri S, Wichmann FA, von Luxburg U (2020). Estimation of perceptual scales using ordinal embedding. J. Vis..

[18] Watson A (1993). Digital Images and Human Vision.

[19] Charrier C, Maloney LT, Cherifi H, Knoblauch K (2007). Maximum likelihood difference scaling of image quality in compression-degraded images. J. Opt. Soc. Am. A.

[20] Ponomarenko N (2009). Tid 2008-a database for evaluation of full-reference visual quality assessment metrics. Adv. Mod. Radioelectron..

[21] Chandler DM (2013). Seven challenges in image quality assessment: Past, present, and future research. ISRN Signal Process..

[22] Koenderink J, Valsecchi M, van Doorn A, Wagemans J, Gegenfurtner K (2017). Eidolons: Novel stimuli for vision research. J. Vis..

[23] Sauer Y, Wahl S, Rifai K (2020). Parallel adaptation to spatially distinct distortions. Front. Psychol..

[24] Brunswik E (1955). Representative design and probabilistic theory in a functional psychology. Psychol. Rev..

[25] Rojo P, Royo S, Ramírez J, Madariaga I (2014). Numerical implementation of generalized Coddington equations for ophthalmic lens design. J. Mod. Opt..

[26] Sitole SP, LaPre AK, Sup FC (2020). Application and evaluation of lighthouse technology for precision motion capture. IEEE Sens. J..

[27] Terada, Y. & Luxburg, U. Local ordinal embedding. In *International Conference on Machine Learning*. pp 847–855 (2014).

[28] Künstle D-E, von Luxburg U, Wichmann FA (2022). Estimating the perceived dimensionality of psychophysical stimuli using a triplet accuracy and hypothesis testing procedure. J. Vis..

[29] Gower JC (1975). Generalized procrustes analysis. Psychometrika.

[30] Breiman L (2001). Random forests. Mach. Learn..

[31] Sauer, Y., Wahl, S., Habtegiorgis, S. W. Realtime blur simulation of varifocal spectacle lenses in virtual reality. In SIGGRAPH Asia. in *Technical Communications, SA ’22 (Association for Computing Machinery* 2022 (New York, NY, USA, 2022).

[32] Rifai K, Wahl S (2016). Specific eye-head coordination enhances vision in progressive lens wearers. J. Vis..

[33] Hutchings N, Irving EL, Jung N, Dowling LM, Wells KA (2007). Eye and head movement alterations in naïve progressive addition lens wearers. Ophthalmic Physiol. Opt..

