# Supplementary material for: An objective measurement approach to quantify the perceived distortions of spectacle lenses
Source: Sci Rep. 2024 Feb 17;14:3967. doi: 10.1038/s41598-024-54368-3 (PMC10874444; doi:10.1038/s41598-024-54368-3)
Supplement: Supplementary file 1 — Supplementary Figures. [file 41598_2024_54368_MOESM1_ESM.pdf]

Supplementary Material

An objective measurement approach to quantify the perceived distortions of spectacle lenses

Yannick Sauer[1], David-Elias Künstle[2], Felix A. Wichmann[3], and Siegfried Wahl[4]

[1,2,3,4]University of Tübingen
[1,4]Carl Zeiss Vision International GmbH
[2]Tübingen AI Center
[1,2]These authors contributed equally to this work.

A Individual subject data

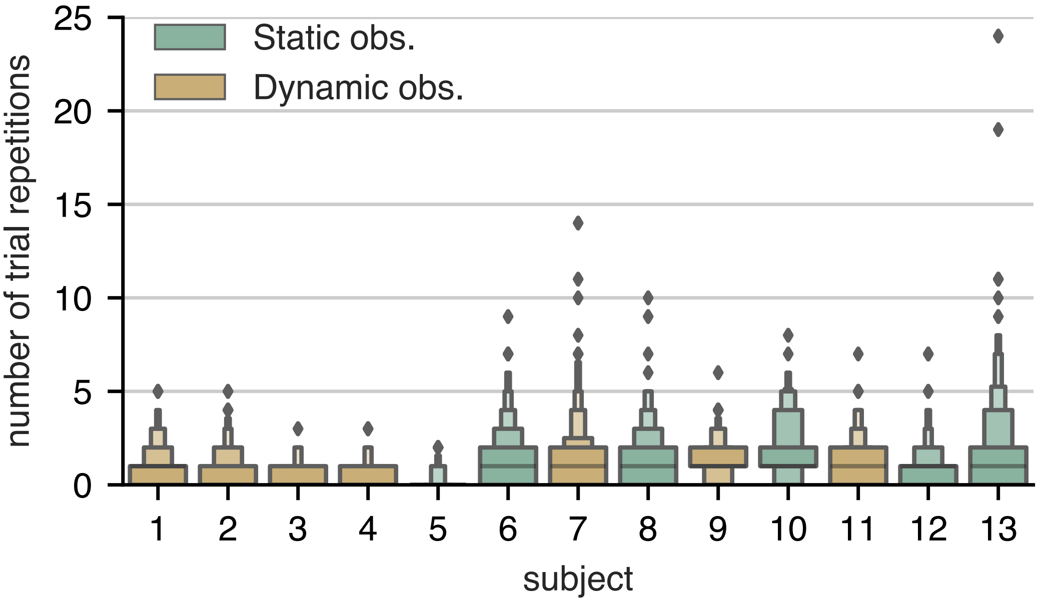

Figure S1: Individual distributions of number of repeated trials per subject.

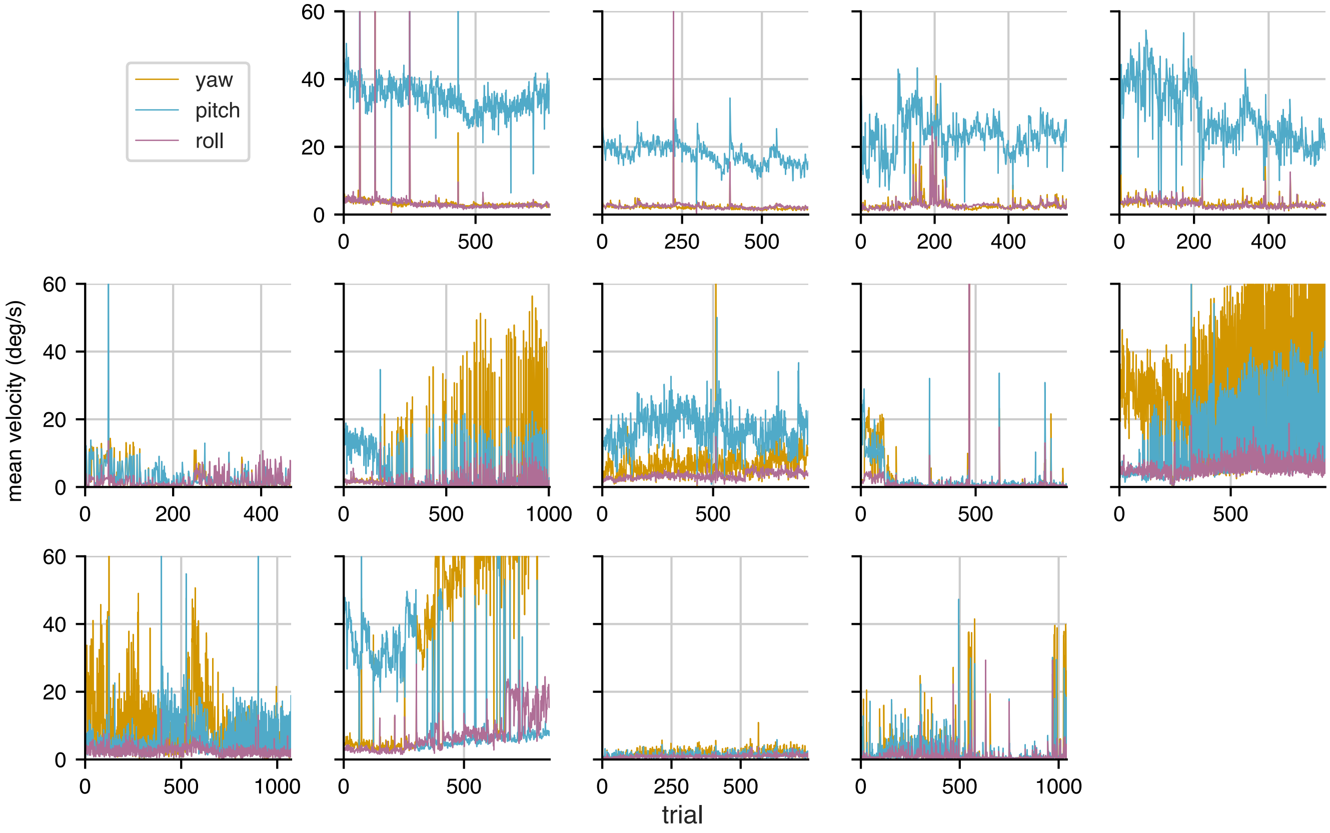

Figure S2: Individual head-movement components for each subject over all trials.

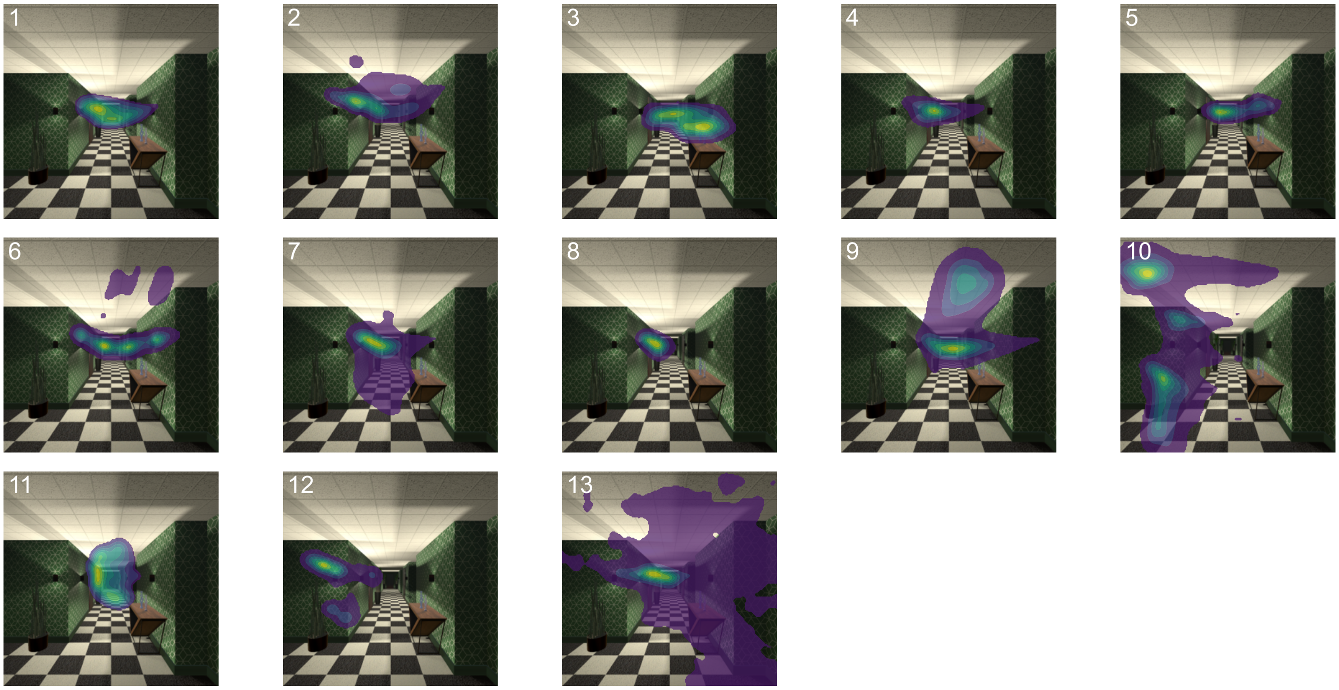

Figure S3: Individual gaze distribution in the scene. The gaze target in the 3D scene is determined by head position and gaze direction. During runtime, the intersection point of the binocular gaze vector and the 3D environment was calculated using a ray cast originating at the headset position. Those 3D points were recorded additionally to all gaze vector samples. From the 3D points we can analyse the 3D gaze distribution in the scene. For visualization, an image of the scene from a fixed viewpoint (the seating position of subjects in the scene) is overlayed with the 3D gaze samples mapped into the scene for the defined viewpoint and smoothed using a Gaussian kernel.

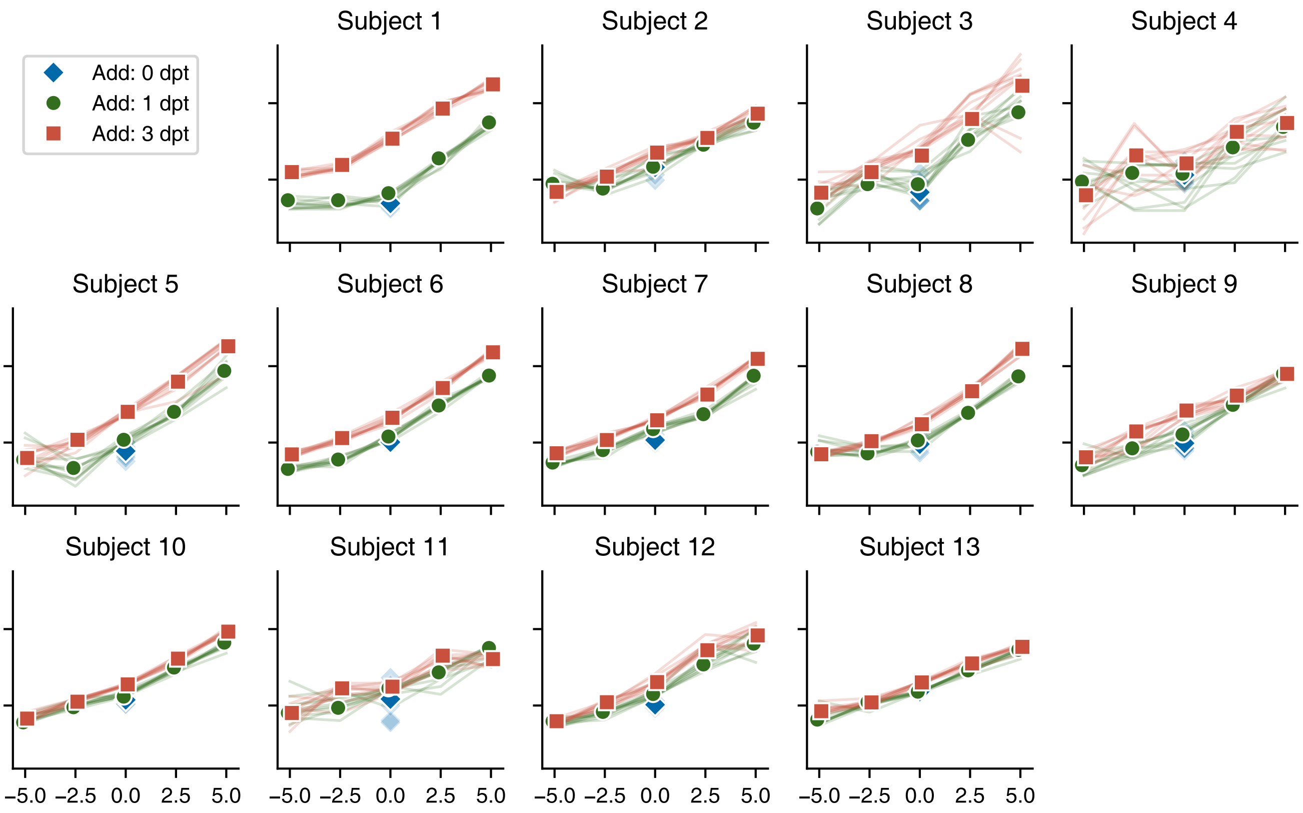

Figure S4: Scales per subject (opaque markers) with bootstrapped variants (transparent markers and lines). The bootstrapped variants are scale estimates based not on all triplets but a random subset of 95% to increase variability. These resamples ought to test the stability of the estimate by simulating how much the scale estimates would change if different triplet questions were asked. For most subjects, these bootstrapped scales are very close to the scale of all triplets, indicating that collecting additional trials would not substantially change the estimate.

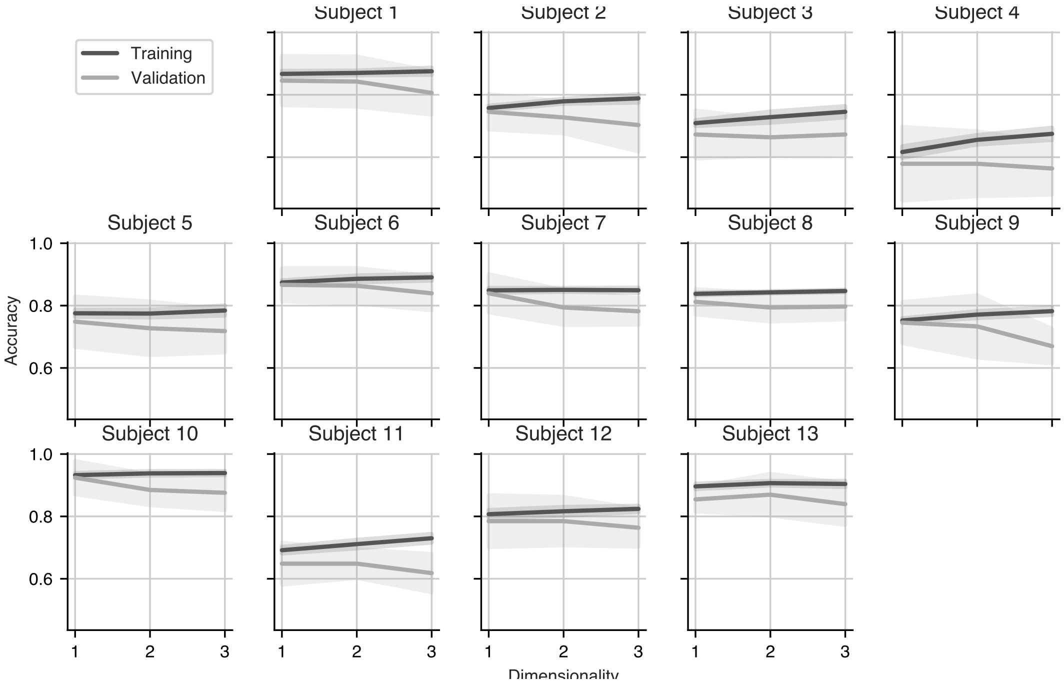

Figure S5: Embedding accuracy depending on embedding dimensionality. Accuracy is the proportion of trials where the response can be predicted from the scale, which was fitted with different dimensionality. The embedding accuracies for all subjects do not increase with the dimensionality, which indicates that a 1D scale already fits the responses sufficiently.
The line and bands show the accuracy mean and standard deviation of 10 so-called cross-validation splits, where the scale was fitted on 90% of the trials ("Training") and validated on the remaining 10% trials. The accuracy of the validation trials ought to approximate the predictive performance for unseen responses. Additionally, the validation accuracy can be understood as an indicator of how consistently subjects respond—subjects 4 and 11 show more inconsistencies than the others.

B Stimuli

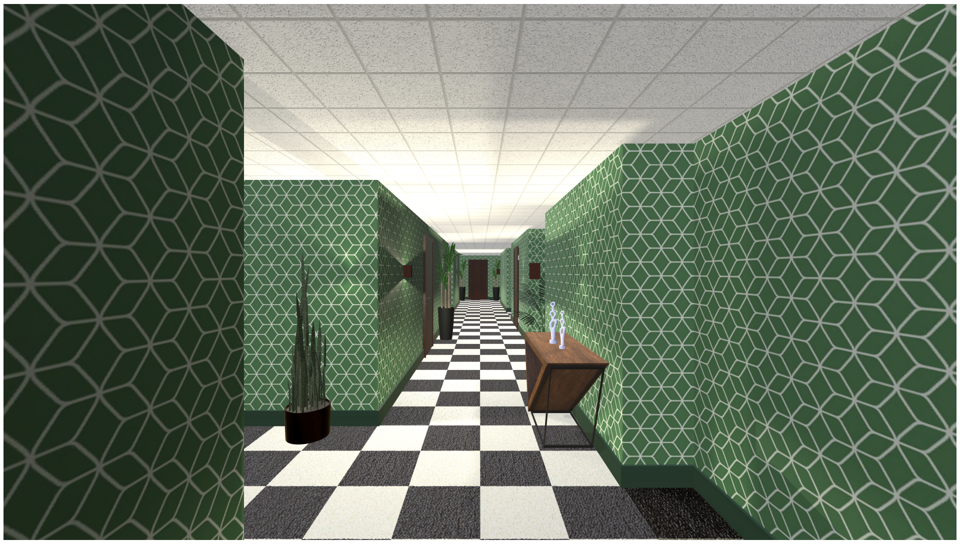

Figure S6: Virtual indoor environment used for the psychophysical experiment in the game engine Unity.

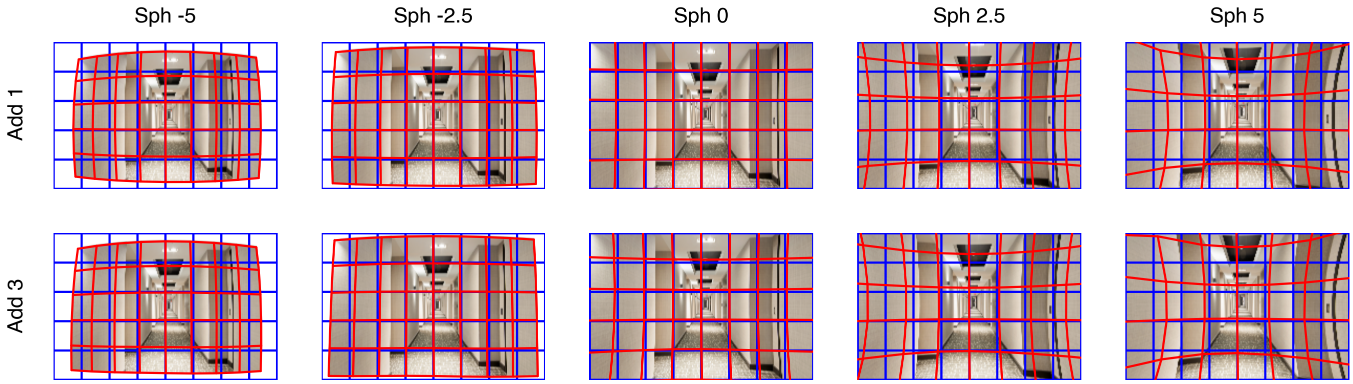

Figure S7: Overview of all ten distortions used in the experiment. The far correction power *Sph* varied between -5 dpt to 5 dpt and the additional power for near vision was 1 dpt or 3 dpt.
